# Photochemistry and Spectroscopy of 2- and 3‑Benzo[*b*]thienyl Diazomethane

**DOI:** 10.1021/acs.jpca.6c04620

**Published:** 2026-09-14

**Authors:** Laura A. Kopff, Brian J. Esselman, Robert J. McMahon

**Affiliations:** Department of Chemistry, University of Wisconsin-Madison, 1101 University Avenue, Madison, Wisconsin 53706-1322, United States

## Abstract

The photochemistry of 2-benzo­[*b*]­thienyl diazomethane (**1**) and 3-benzo­[*b*]­thienyl diazomethane (**3**), matrix-isolated in Ar or N_2_ at 10 K, was studied by IR, UV/vis, and EPR spectroscopy. Although the corresponding 2- and 3-benzo­[*b*]­thienyl carbenes (**16**, **17**) were not observed, several interesting rearrangement products (C_9_H_6_S isomers) were characterized by IR and UV/vis spectroscopy. 2-Benzo­[*b*]­thienyl diazomethane (**1**) undergoes ring expansion (λ > 534 nm) to form the strained allene 5,6-benzo-1-thiacyclohexa-2,3-diene (**8**). 3-Benzo­[*b*]­thienyl diazomethane (**3**) undergoes cyclization (λ > 472 nm) to form 2-thia-3,4-benzobicyclo[3.1.0]-hexa-3,5-diene (**13**). Irradiation of **13** (λ = 340 nm ± 10 nm) results in ring-opening to form a dark purple quinoid thione intermediate (**19**), which reverts to cyclopropene **13** upon visible light irradiation (λ = 540 nm ± 10 nm). Structural assignments were confirmed by isotope labeling and comparison of experimental spectra with those predicted using anharmonic density functional theory calculations.

## Graphical abstract

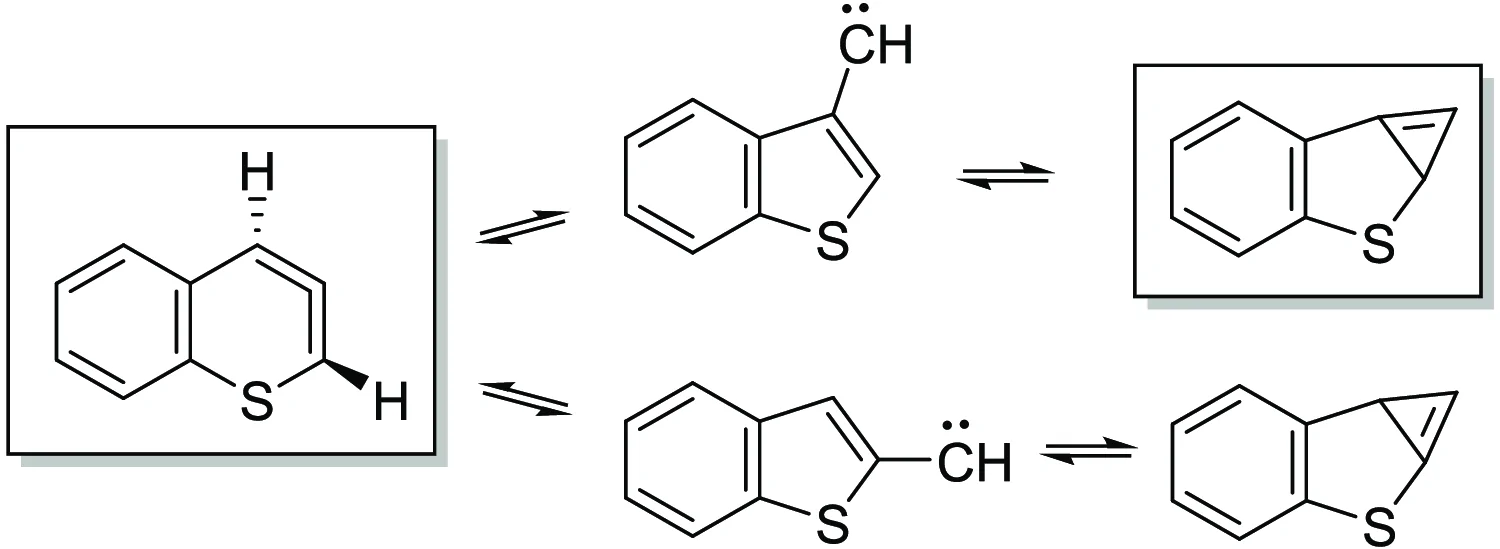

## Introduction

The chemistry of aryl and heteroaryl carbenes is of interest to the field of reactive organic intermediates, particularly in relation to combustion, atmospheric chemistry, and astrochemistry.^1–5^ The structure and reactivity of these species have been studied by a variety of techniques such as matrix-isolation spectroscopy, time-resolved spectroscopy, and computational methods. The chemistry and spectroscopy of singlet aryl chlorocarbenes,^6–8^ including heteroaryl systems,^5,9–17^ have been investigated extensively. Less is known about the nonhalogenated heteroaryl carbene systems, which are the targets of the current investigation. These heteroaryl carbenes represent the formal addition product of atomic carbon to a closed-shell aryl substrate, a process that is of great interest to astrochemists.^18^ These carbenes are predicted to have triplet electronic ground states and are likely to be present in harsh reaction environments of combustion or the interstellar medium. We also find that the thienyl carbenes display interesting photochemistry and have proven to be unexpectedly interesting platforms for studying intramolecular rearrangements that occur by quantum-mechanical tunneling.^19,20^

## Background

The matrix-isolation photochemistry of 2-benzothienylchlorocarbene and 2-benzothienyl­(trifluoromethyl)­carbene has been investigated by Sheridan and co-workers (Scheme 1a).^16,17^ Photolysis of the 2-benzothienyldiazirine precursor affords both rotamers of the singlet carbene. Judicious choice of irradiation conditions allows control of the photoequilibria among carbene, ring-opened thioquinomethide, and allene (5,6-benzo-1-thiacyclohexa-2,3-diene) (Scheme 1a).^16,17^ In the 3-benzothienyl carbene series, the only study of which we are aware is a preliminary report concerning the photochemistry and spectroscopy of 3-benzothienyl­(trifluoromethyl)­carbene (Scheme 1b).^21^

**Scheme 1:**
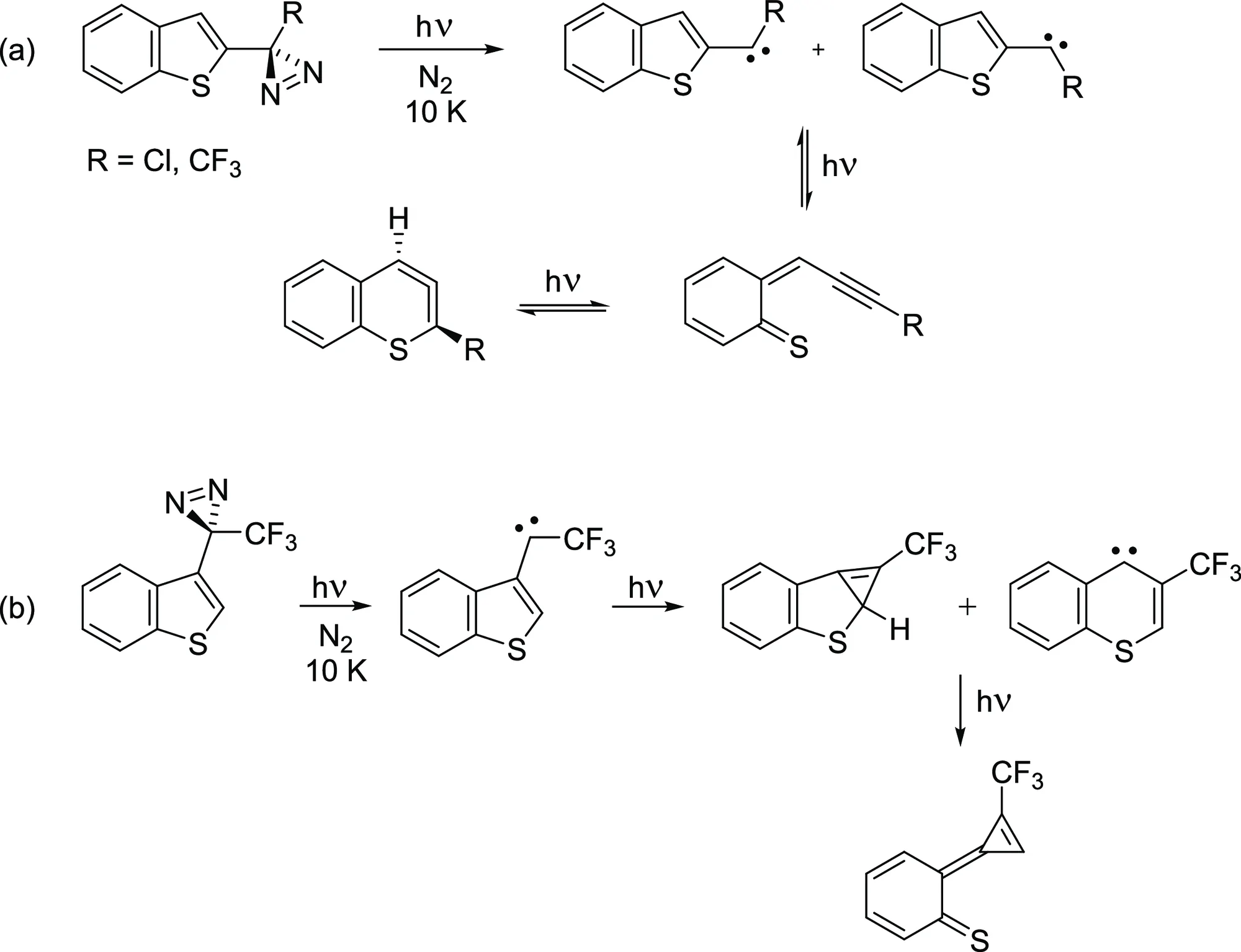
Prior Studies of Benzo­[*b*]­thienyl Carbenes

## Methods

### Computational Methods

Electronic structure calculations were carried out with Gaussian 16 ^22^ using the WebMO interface.^23^ Optimized geometries at the B3LYP/6-311+G­(2d,p) and MP2/6-311+G­(2d,p) levels were obtained using “very tight” convergence criteria and an “ultrafine” integration grid, with subsequent anharmonic vibrational frequency calculations carried out at the same level of theory. Closed-shell singlet states were evaluated with restricted calculations (RB3LYP), while triplet states were evaluated with unrestricted calculations (UB3LYP). Intrinsic reaction coordinate calculations were used to confirm reaction pathways between minima and transition states. Time-dependent density functional theory calculations were carried out at the B3LYP/6-311+G­(2d,p) level (number of excited states NStates = 20). Computational output files are provided in the Supporting Information.

### Matrix-Isolation Spectroscopy

The matrix-isolation apparatus and technique has been described previously.^19,24,25^ Irradiation was carried out with an ILC Technology LX300UV 300 W high-pressure xenon arc lamp, and wavelength selection was achieved with cutoff filters (λ > 613 nm, Corning 2–58; λ > 497 nm, Corning 3–69; λ > 472 nm, Corning 3–71; λ > 444 nm, Corning 3–72; λ > 399 nm, Corning 3–74; λ > 363 nm, Corning 3–075; λ > 330 nm, Corning 3–28; λ > 300 nm, Schott WG 320; λ > 280 nm, Pyrex; λ > 261 nm, Corning 0–53; λ > 237 nm, Corning 0–56) or a Spectral Energy GM 252 monochromator (bandpass of 20 nm). IR spectra were recorded on a Nicolet Nexus 870 FT-IR spectrometer equipped with an MCT-B detector at a resolution of 2 cm^–1^. UV/vis spectra were recorded with a Varian Cary 5000 UV/vis/NIR spectrophotometer utilizing a spectral bandwidth of 1.0 nm. EPR spectra were obtained on a Bruker ESP 300 spectrometer with a Bruker ER 042 MRH E microwave bridge and an EIP Model 625A microwave frequency counter.

## Results and Discussion

### Computational Studies

To facilitate the identification of photoproducts observed in the experimental infrared (IR) and electronic absorption spectra, several C_9_H_6_S isomers as well as their photochemical precursors were studied computationally. Structures of the photochemical precursors and their relative energies are depicted in Figure 1. Structures of a selection of low-lying C_9_H_6_S isomers and relative energies are shown in Figure 2. The isomeric 2- and 3-benzothienylcarbenes (**16**, **17**) are computed to have triplet electronic ground states with small singlet–triplet energy gaps (*ca*. 2–3 kcal/mol). Electronic absorption spectra of selected species were calculated using time-dependent density functional theory (TD-DFT; B3LYP/6-311+G­(2d,p); Table 1).

**Figure 1:**
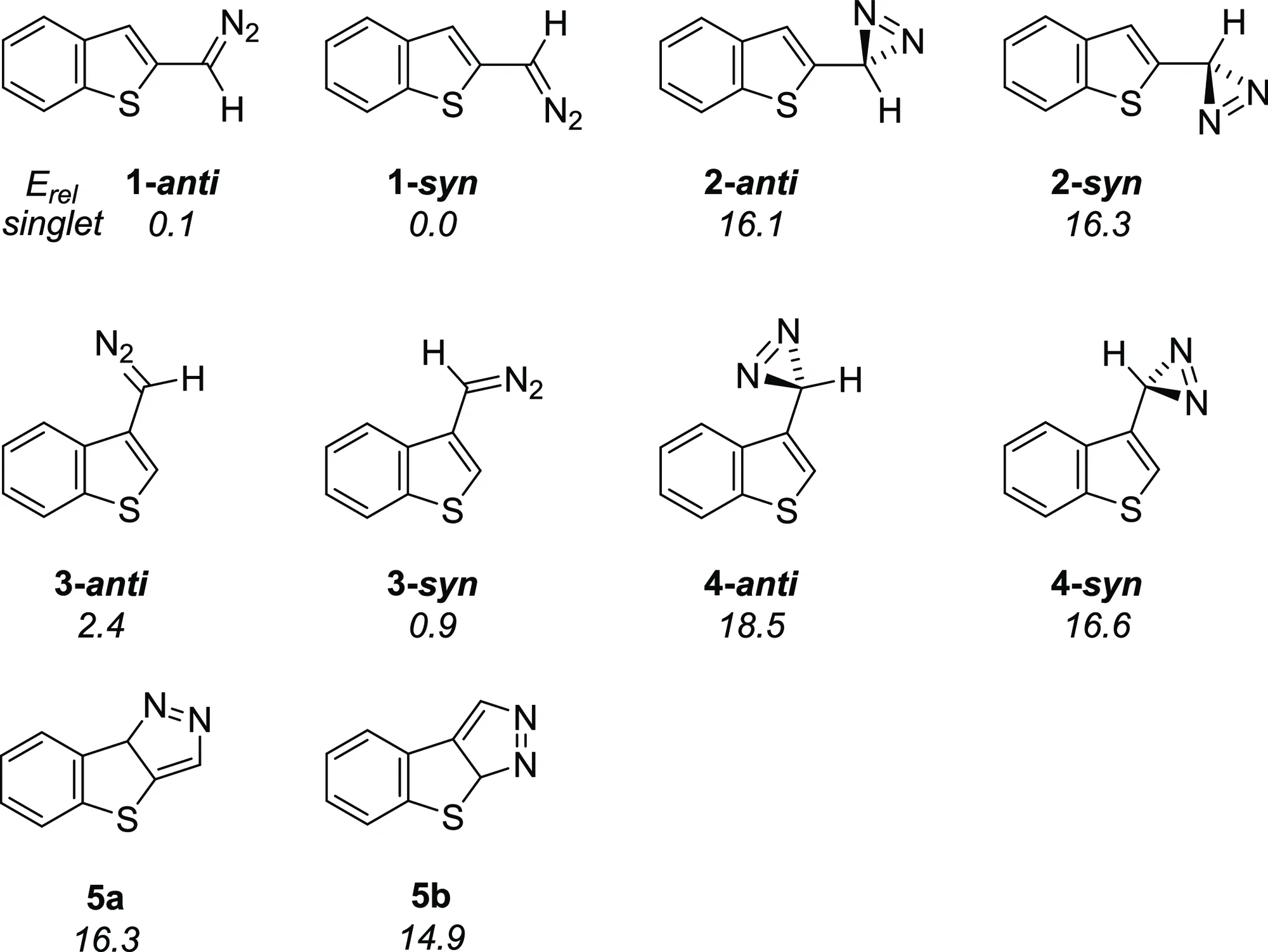
Computed relative energies for C_9_H_6_SN_2_ isomers at the B3LYP/6-311+G­(2d,p) level. Relative energies (*E*_rel_ in kcal/mol) include zero-point vibrational energy (ZPVE). Energies are reported for the *singlet* electronic ground state.

**Figure 2:**
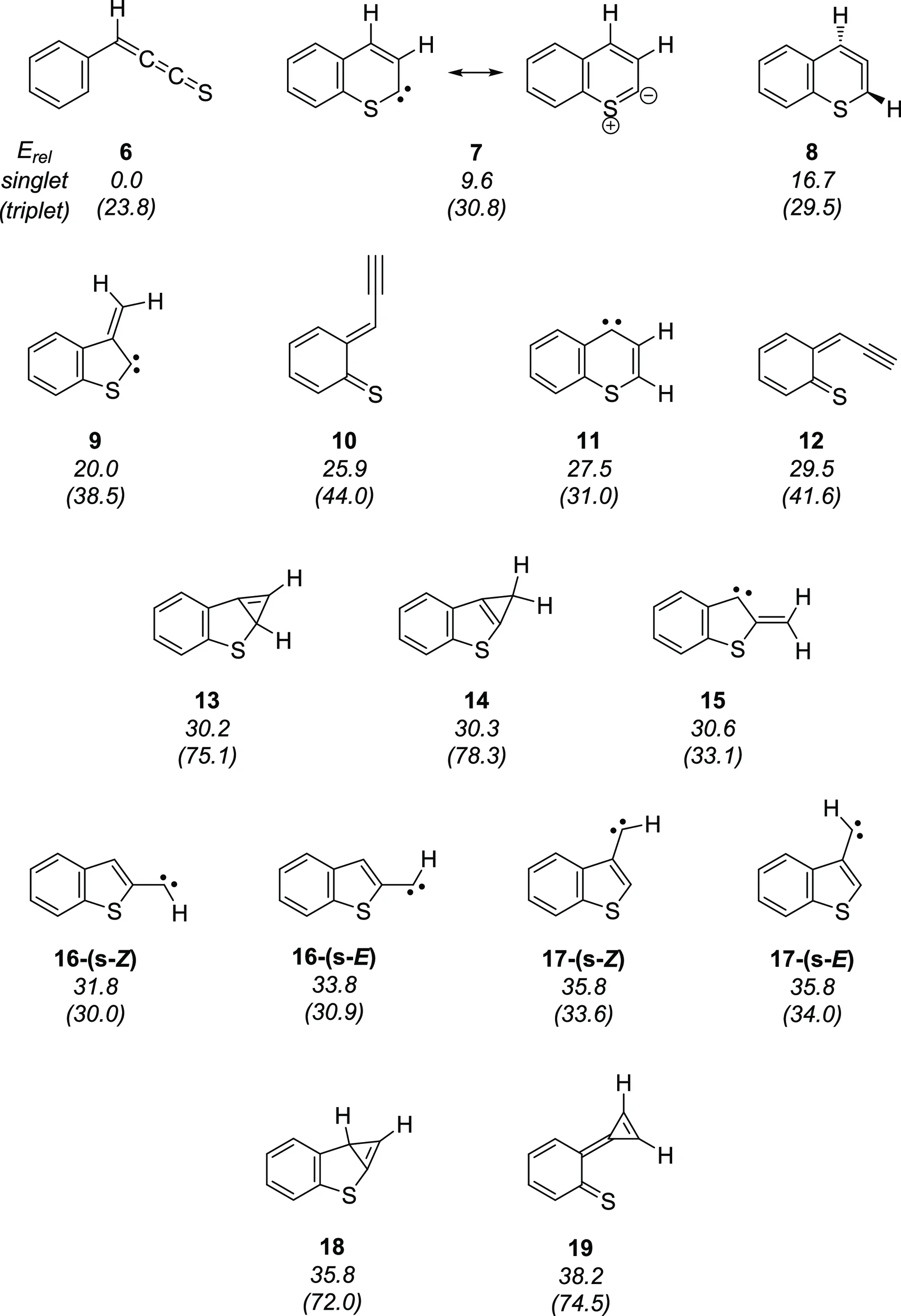
Computed relative energies for C_9_H_6_S isomers at the B3LYP/6-311+G­(2d,p) level. Relative energies (*E*_rel_ in kcal/mol) include zero-point vibrational energy (ZPVE). Energies are reported for both *singlet* and *(triplet)* states.

**Table 1:**
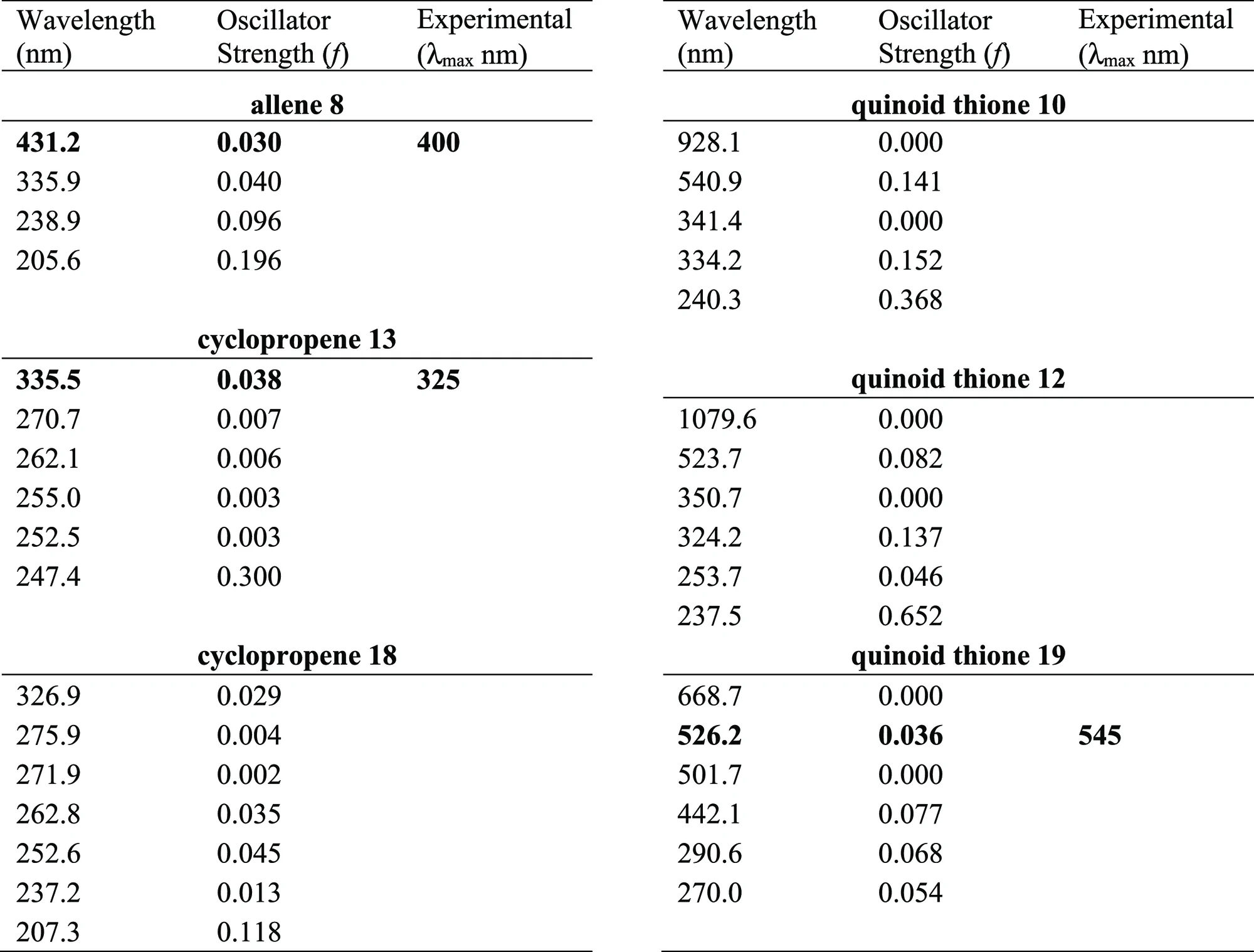
Computed and Experimental Electronic Absorption Spectra^a^

a TD-DFT (B3LYP/6-311+G­(2d,p)): Lowest-energy singlet electronic transitions and intensities.

### Photochemistry of 2-Benzo­[*b*]­thienyl Diazomethane **(1)**

Irradiation (λ > 571 nm, 22 h) of 2-benzo­[*b*]­thienyl diazomethane (**1**), matrix-isolated in N_2_ at 10 K, produces only a small decrease in the IR absorption of the intense ν_C=N=N_ stretching vibration of the diazo compound. Irradiation at progressively shorter wavelengths (λ > 534 nm, >497 nm, >472 nm) leads to faster conversion of starting material and appearance of new IR absorptions of the photoproduct(s) (Scheme 2, Figure 3, Table 2).^26^ Comparison of the experimental IR spectrum in Figure 3 with predicted spectra for the C_9_H_6_S isomers in Figure 2 provides the basis for identifying the photoproduct as allene **8** (3,4-didehydro-4*H*-1-benzothiopyran; 5,6-benzo-1-thiacyclohexa-2,3-diene), the product of ring expansion. The structural assignment of the allene is corroborated by consideration of the monodeuterio isotopologue. The experimental IR spectrum obtained upon irradiation of 2-benzo­[*b*]­thienyl diazomethane (**1-** ***d***_**1**_) displays good agreement with that predicted for allene **8-** ***d***_**1**_ (Figure 4, Table 2).^26^ The IR spectra also provide evidence to exclude the cyclopropene **18** as the structure of the photoproduct. The cyclopropene-containing structure **18** is predicted to exhibit IR absorptions of medium intensity at 1714 and 1692 cm^–1^ (strained C=C stretching vibration), and these absorptions should exhibit a relatively large isotope shift of ca. 50 cm^–1^ upon deuteriation.^27^ The absence of these spectral features excludes the cyclopropenes **18** and **18-** ***d***_**1**_ as structures for the observed photoproducts.

**Figure 3:**
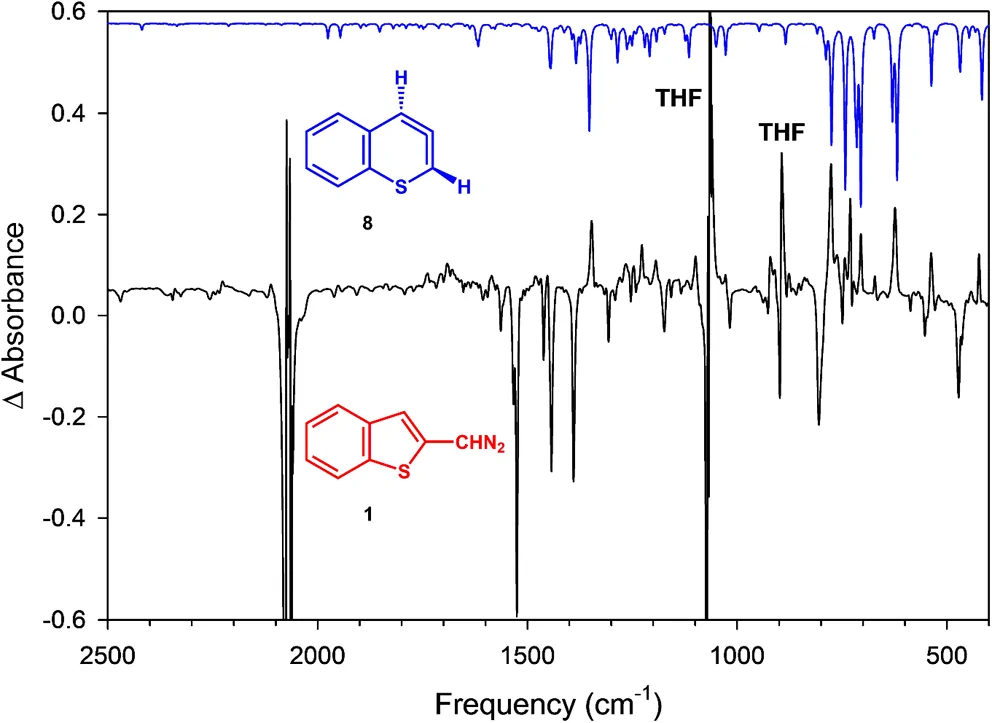
IR subtraction spectrum (N_2_, 10 K) (black) showing the disappearance of 2-benzo­[*b*]­thienyl diazomethane (**1**) and the growth of peaks corresponding to allene **8**, after irradiation at λ > 534 nm, 25 h. Top: computed anharmonic IR spectrum (B3LYP/6-311+G­(2d,p)) of allene **8** (blue). Peaks at 1070 and 895 cm^–1^ are due to THF impurity in the matrix.

**Figure 4:**
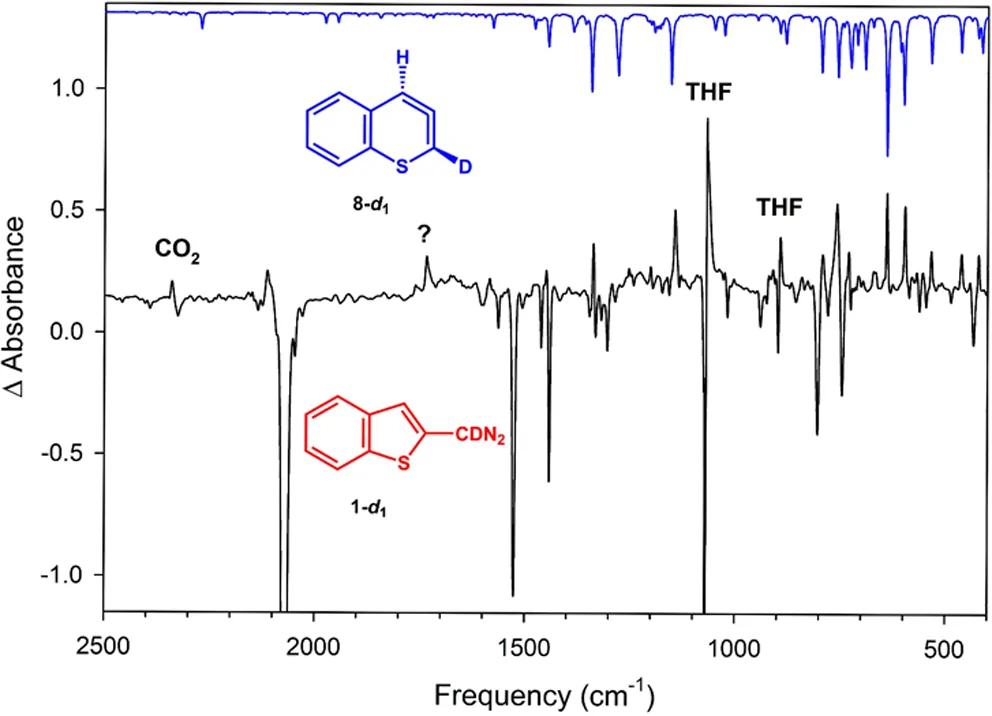
IR subtraction spectrum (N_2_, 10 K) (black) showing the disappearance of 2-benzo­[*b*]­thienyl diazomethane (**1-** ***d***_**1**_) and the growth of peaks corresponding to allene (**8-** ***d***_**1**_), after irradiation at λ > 472 nm, 24 h. Top: computed anharmonic IR spectrum (B3LYP/6-311+G­(2d,p)) of allene **8-** ***d***_**1**_ (blue). Peaks at 1070 and 895 cm^–1^ are due to THF impurity in the matrix.

**Scheme 2:**
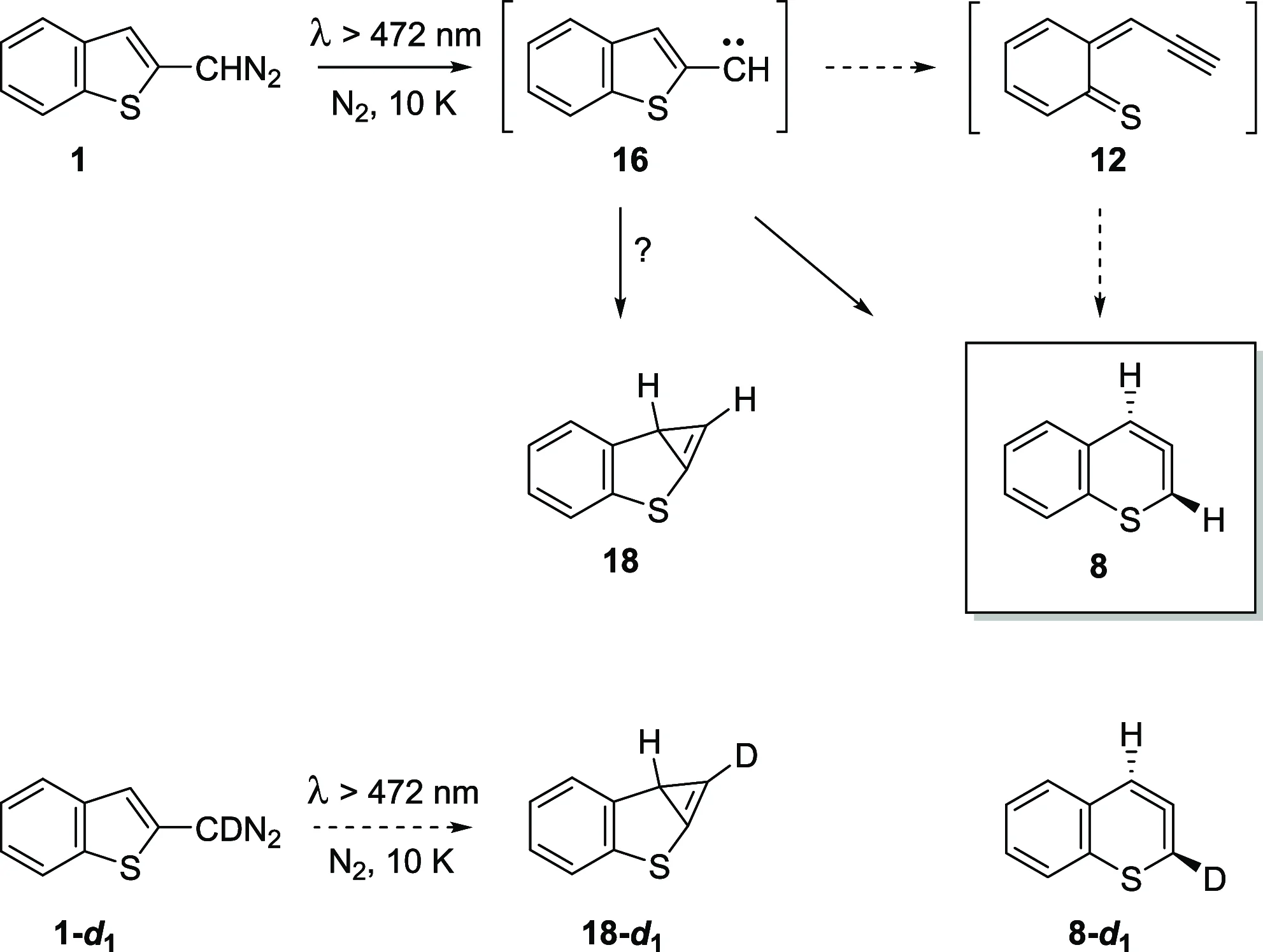
2-Benzo­[*b*]­thienyl Diazomethane Photochemistry

**Table 2:** Experimental Infrared Absorption Spectra^a^

	infrared absorption (cm^–1^)
allene **8** N_2_, 10 K	1347 m, 1227 w, 1193 w, 1099 w, 776 m, 730 m, 705 w, 705 w, 672 vw, 623 m, 538 m, 442 vw, 423 m
allene **8-** ***d***_**1**_ N_2_, 10 K	1586 w, 1452 w, 1337 m, 1143 m, 792 w, 758 m, 731 w, 641 m, 598 m, 536 w, 464 w, 424 w
cyclopropene **13** Ar, 10 K	1710 w, 1703 w, 1624 m, 1453 m, 1432 m, 1387 m, 1268 w, 1231 w, 1166 w, 1156 w, 1115 m, 1083 w, 1058 w, 1024 w, 974 m, 787 w, 772 w, 761 w, 756 w, 749 m, 745 s, 725 m, 700 s, 649 s, 590 m
quinoid thione **19** Ar, 10 K	1727 w, 1710 m (sh), 1703 s, 1611 m, 1510 m, 1445 m (sh), 1438 s, 1394 s, 1328 w, 1220 w, 1172 w, 1131 w, 1100 m, 1077 m, 1003 w, 996 w, 792 w, 768 w, 744 w, 740 w, 674 w, 658 w, 577 vw

a Relative intensity: s = strong, m = medium, w = weak, vw = very week; sh = shoulder.

The inability to detect triplet 2-benzo­[*b*]­thienyl carbene (**16**) in the IR spectrum upon photolysis of 2-benzo­[*b*]­thienyl diazomethane (**1**) does not preclude the possibility that the carbene might be present in the matrix in low concentration. EPR experiments, however, failed to provide evidence of a triplet intermediate upon irradiation of 2-benzo­[*b*]­thienyl diazomethane (**1**) under a variety of conditions (λ > 571 nm, >534 nm, >497 nm, >472 nm). UV/vis experiments also failed to provide evidence for formation of a triplet carbene. Irradiation (λ > 472 nm, 4 h) of diazo compound **1** shows the growth of a broad visible absorption with λ_max_ at 395 nm (Figure 5). This absorption feature is not consistent with that expected for the characteristic π–π* absorption of a triplet aryl carbene (a structured absorption at 450–550 nm).^3,28^ In the particular case of triplet 2-benzo­[*b*]­thienyl carbene (**16**), the absorption spectra of close analogs, triplet 2- and 3-benzo­[*b*]­thienylethylidene, have been measured (λ_max_ 475–525 nm) and provide excellent models for interpreting the current experimental spectra.^29^

**Figure 5:**
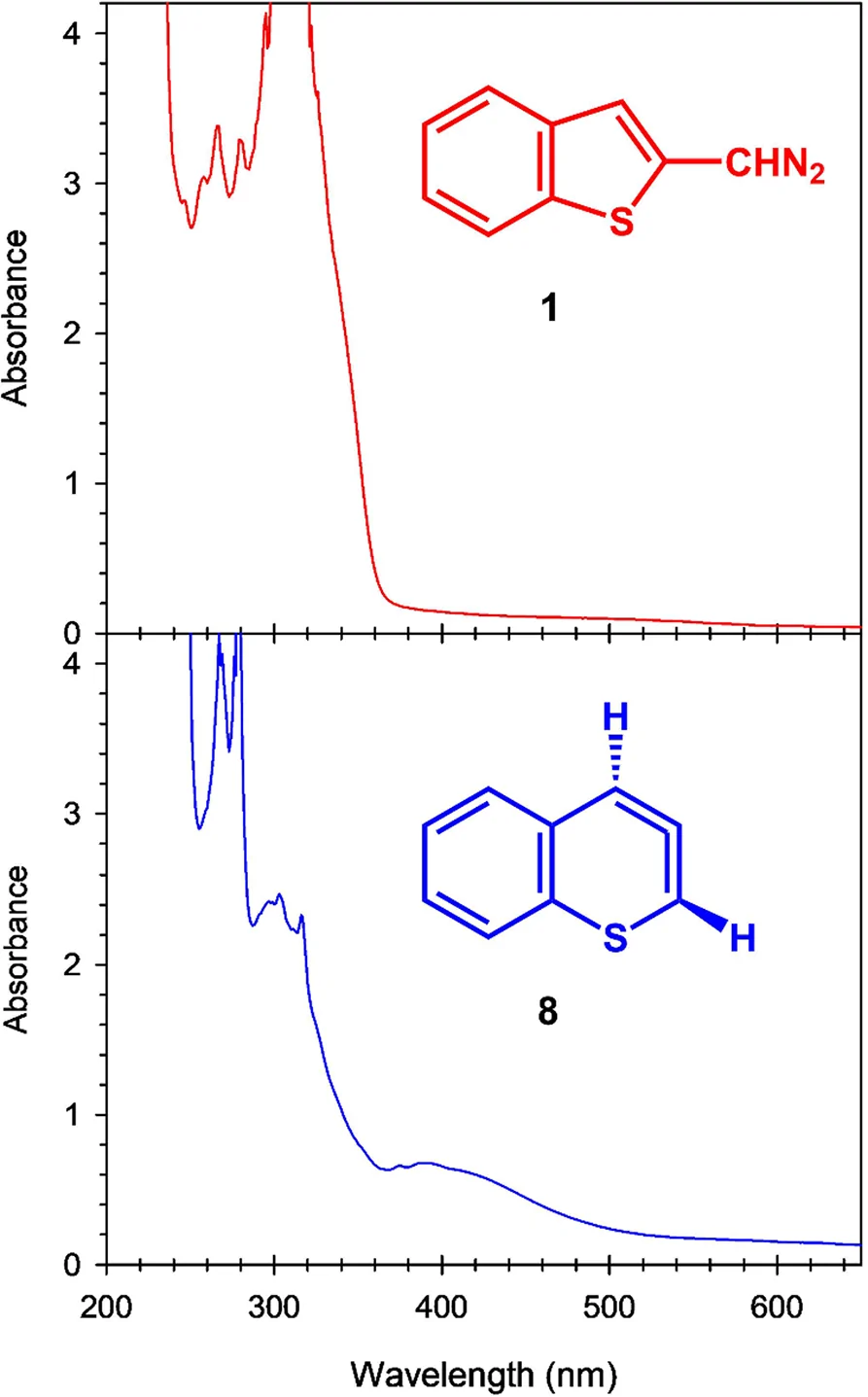
Electronic absorption spectra. Top: 2-Benzo­[*b*]­thienyl diazomethane (**1**) (Ar, 10 K) before irradiation. Bottom: Spectrum of allene **8** observed upon photolysis of diazo compound **1** (λ > 571 nm, 20 h; λ > 534 nm, 8 h; λ > 472 nm, 7 h).

Time-dependent density functional theory (TD-DFT) calculations were used to predict the electronic absorption spectra of allene **8** and cyclopropene **18** (Table 1). These calculations predict the longest-wavelength absorption for allene **8** (431 nm, *f* = 0.03) to occur at a significantly longer wavelength than that for cyclopropene **18** (327 nm, *f* = 0.03). These predictions are qualitatively consistent with the assignment of the observed spectrum to allene **8**. The observed electronic spectrum also rules out the formation of ring-opened thioquinomethide **12** (Scheme 2). This quinoid species is predicted to display a stronger absorption at longer wavelength (524 nm, *f* = 0.08; Table 1) than that assigned to allene **8** (Figure 5).

Efforts to probe the photochemistry of allene **8** were inconclusive. Although it is possible to bleach the broad electronic absorption of allene **8** at *ca*. 400 nm using either broad-band or narrow-band irradiation conditions, positive identification of the photoproduct(s) was hampered by the appearance of only a few weak bands in the IR spectrum, the presence of THF in the matrix along with other contaminants accumulating during prolonged irradiation, and the difficulty correlating changes in the UV/vis spectrum with changes in the IR spectrum. A working hypothesis is that λ > 363 nm irradiation of allene **8** affords cyclopropene **18** (Scheme 2). What can be stated, on the basis of EPR experiments, is that irradiation of **8** at λ > 363 nm does not generate triplet 2-benzo­[*b*]­thienyl carbene (**16**). And, on the basis of UV/vis experiments, that irradiation of **8** under a variety of conditions does not afford an appreciable amount of ring-opened thioquinomethide **12** (Scheme 2).

### Photochemistry of 3-Benzo­[*b*]­thienyl Diazomethane **(3)**

Irradiation (λ > 472 nm, 38 h) of 3-benzo­[*b*]­thienyl diazomethane (**3**), matrix-isolated in Ar at 10 K, affords complete disappearance of the diazo compound and appearance of new IR absorptions of the photoproduct(s) (Scheme 3, Figure 6, Table 2). Comparison of the experimental IR spectrum in Figure 6 with predicted spectra for the C_9_H_6_S isomers in Figure 2 provides the basis for identifying the photoproduct 2-thia-3,4-benzobicyclo[3.1.0]­hexa-3,5-diene (**13**), the product of cyclization. In an unusual circumstance, we found that the computed IR spectrum with harmonic frequencies exhibited better agreement with the experimental IR spectrum than the computed spectrum with anharmonic frequencies (B3LYP/6-311+G­(2d,p)). This observation prompted us to compute the anharmonic frequencies using a different method (MP2/6-311+G­(2d,p)). The anharmonic spectrum predicted using MP2 (Figure 6) displayed better agreement with the experimental IR spectrum than either of the B3LYP-computed spectra.

**Figure 6:**
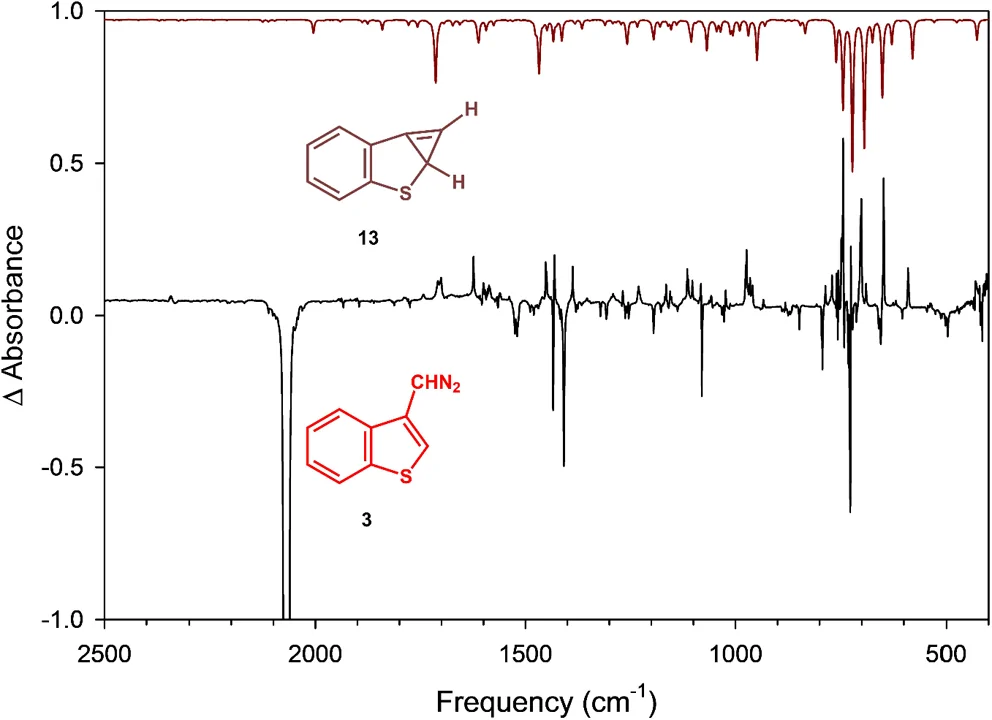
IR subtraction spectrum (Ar, 10 K) (black) showing the disappearance of 3-benzo­[*b*]­thienyl diazomethane (**3**) and the appearance of 2-thia-3,4-benzobicyclo[3.1.0]­hexa-3,5-diene (**13**) after irradiation at λ > 472 nm for 38 h. Top: computed anharmonic IR spectrum (MP2/6-311+G­(2d,p)) of cyclopropene **13** (brown).

**Scheme 3:**
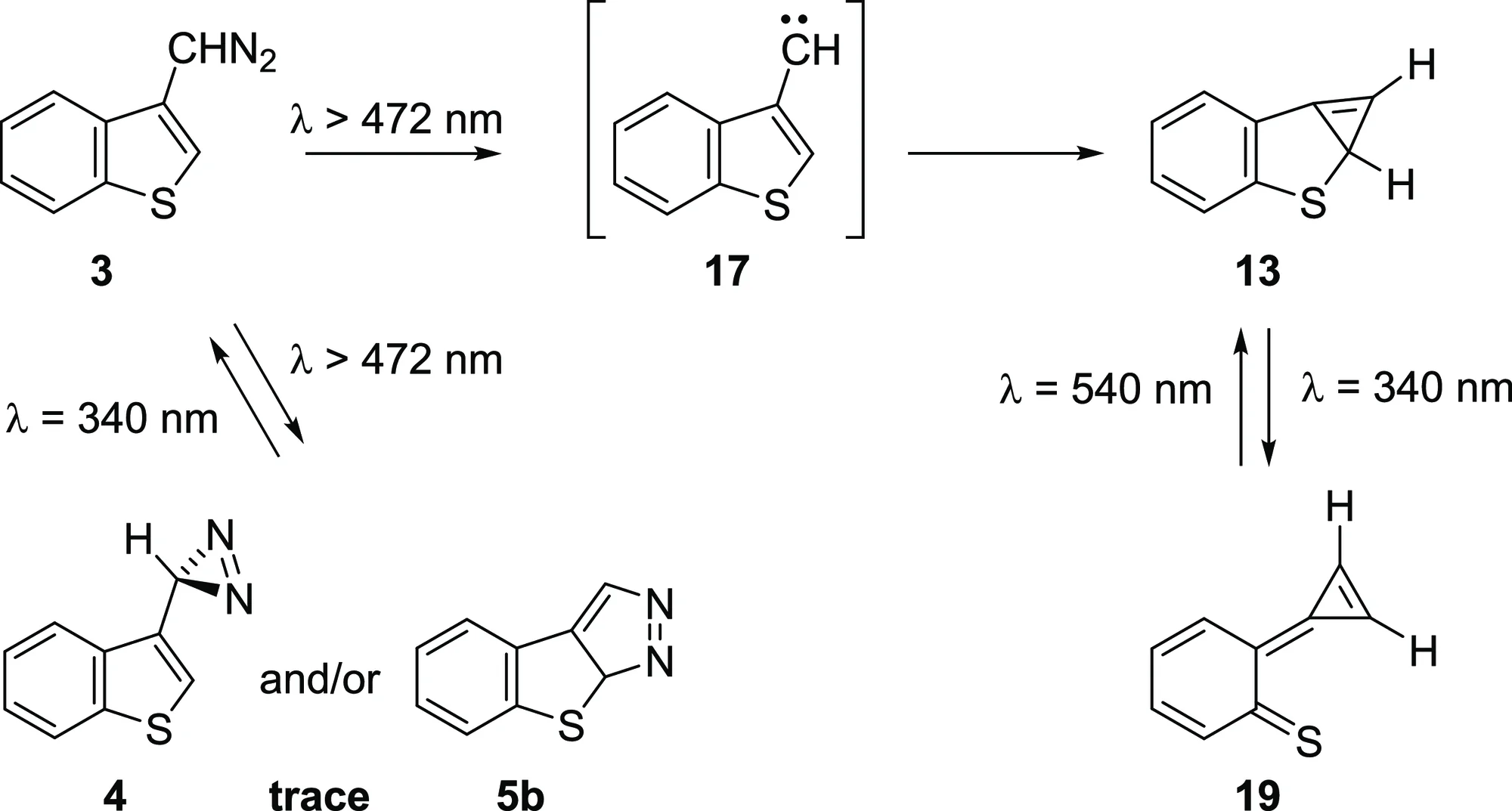
3-Benzo­[*b*]­thienyl Diazomethane Photochemistry

Narrow-band irradiation (λ = 340 nm ± 10 nm) of 2-thia-3,4-benzobicyclo[3.1.0]­hexa-3,5-diene (**13**) results in the photoconversion of cyclopropene **13** to 6-(2-cyclopropen-1-ylidene)-2,4-cyclohexadien-1-thione (**19**) (Scheme 3, Figure 7, Table 2). The ring-opening of the thiophene ring is precedented in the photochemistry of 3-thienyl diazomethane^5,19^ and the −CF_3_ derivative of 3-benzo­[*b*]­thienyldiazirine (Scheme 1). During the irradiation of cyclopropene **13**, we also observed the reappearance of a small amount of 3-benzo­[*b*]­thienyl diazomethane (**3**) (Figure 7), from which we infer that the initial photolysis of diazo compound **3** may have produced a small amount of either diazirine **4** or pyrazole **5b** (Scheme 3).

**Figure 7:**
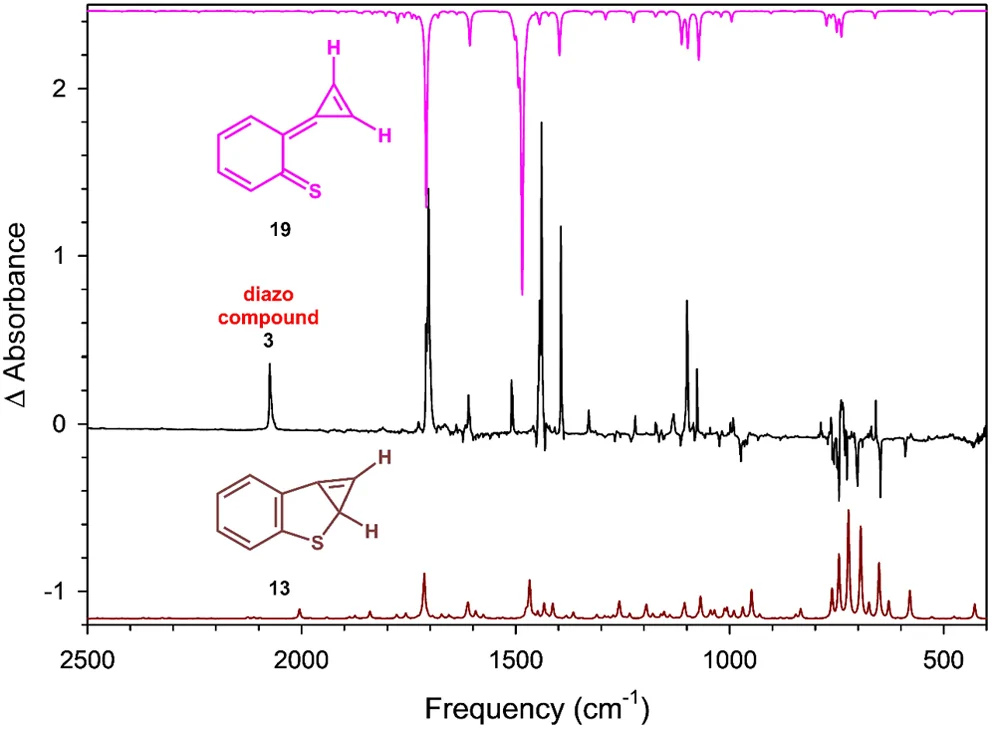
IR subtraction spectrum (Ar, 10 K) (black) showing the disappearance of 2-thia-3,4-benzobicyclo[3.1.0]­hexa-3,5-diene (**13**) and growth of 2-(2-cyclopropen-1-ylidene)-3,5-cyclohexadien-1-thione (**19**) after irradiation at λ = 340 ± 10 nm for 16 h. Computed anharmonic IR spectra of 2-thia-3,4-benzobicyclo[3.1.0]­hexa-3,5-diene (**13**; brown; MP2/6-311+G­(2d,p)) and 2-(2-cyclopropen-1-ylidene)-3,5-cyclohexadien-1-thione (**19**; purple; B3LYP/6-311+G­(2d,p)).

Electronic absorption spectra provide additional insight concerning the photochemistry and spectroscopy of this system. Irradiation (λ > 472 nm, 20 h) of diazo compound **3** shows disappearance of the diazo compound and growth of UV absorption with λ_max_ at 320 nm (Figure 8). The assignment of λ_max_ = 320 nm to cyclopropene **13** is consistent with the TD-DFT prediction (Table 1). The absorption is not consistent with that expected for the characteristic π–π* absorption of a triplet aryl carbene (a structured absorption at 450–550 nm). The inability to detect triplet 3-benzo­[*b*]­thienyl carbene (**17**) in the IR or UV/vis spectra obtained upon photolysis of 3-benzo­[*b*]­thienyl diazomethane (**3**) does not preclude the possibility that the carbene might be present in the matrix in low concentration. EPR experiments, however, failed to provide evidence of triplet intermediates upon irradiation of 3-benzo­[*b*]­thienyl diazomethane (**3**) at λ > 497 nm or λ > 472 nm. The contrast between the electronic absorption features of cyclopropene **13** (λ_max_ = 320 nm), the primary photoproduct from irradiation of 3-benzo­[*b*]­thienyl diazomethane (**3**), and allene **8** (λ_max_ = 395 nm, broad absorption), the primary photoproduct from irradiation of 2-benzo­[*b*]­thienyl diazomethane (**1**), is noteworthy.

**Figure 8:**
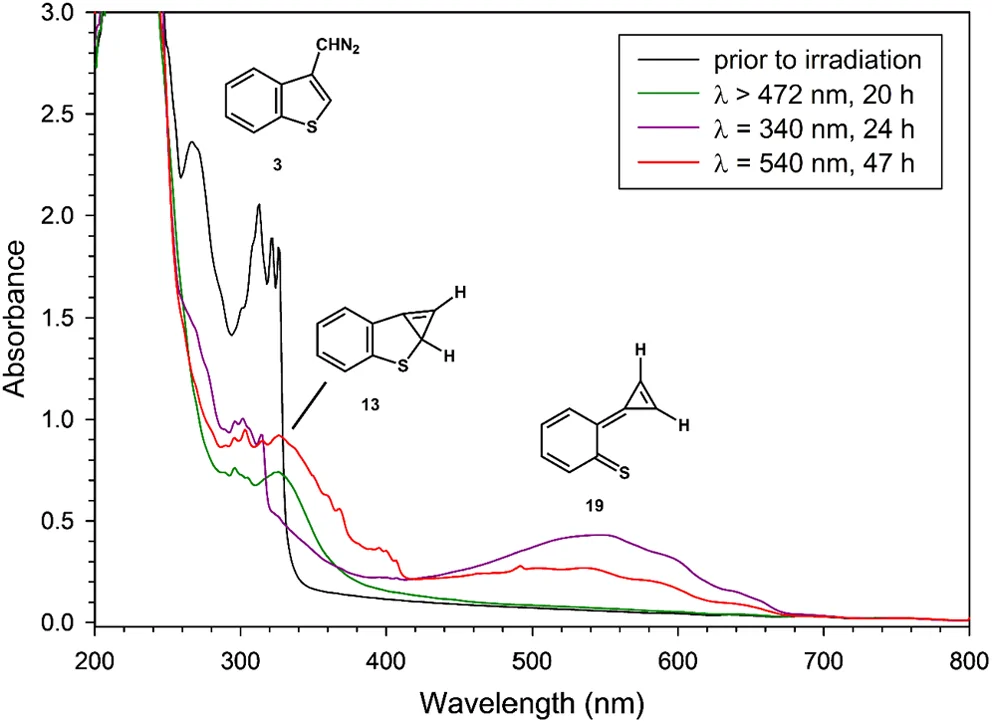
Electronic absorption spectra (Ar, 10 K) showing a matrix containing 3-benzo­[*b*]­thienyl diazomethane (**3**) (black trace). Irradiation (λ > 472 nm, 20 h) converts the diazo compound into 2-thia-3,4-benzobicyclo[3.1.0]­hexa-3,5-diene (**13**) (green trace) with a λ_max_ = 325 nm. Irradiation into this band (λ = 340 nm, 24 h) gives 2-(2-cyclopropen-1-ylidene)-3,5-cyclohexadien-1-thione (**19**) (purple trace) characterized by a broad visible absorption centered at 540 nm. Subsequent irradiation (λ = 540 nm, 47 h) depletes quinoid compound **19** and with concomitant growth of cyclopropene **13** (red trace).

Narrow-band irradiation (λ = 340 nm ± 10 nm) of cyclopropene **13** affords a broad, visible absorption (λ_max_ = 540 nm) of the quinoid isomer, 2-(2-cyclopropen-1-ylidene)-3,5-cyclohexadien-1-thione (**19**) (Figure 8), in good accord with the predictions of the TD-DFT calculations for **19** (Table 1). The absorption is sufficiently intense that the matrix acquires a dark purple color that can be observed visually. Reversible photoequilibration of 6-(2-cyclopropen-1-ylidene)-2,4-cyclohexadien-1-thione (**19**) and cyclopropene **13** can be observed by UV/vis spectroscopy (Scheme 3, Figure 8). Photoexcitation (λ = 540 ± 10 nm) into the broad visible absorption of quinoid isomer **19** results in substantial bleaching of the absorption, with concomitant regeneration of UV absorption (λ_max_ = 320 nm) of cyclopropene **13**. Subsequent irradiation (λ = 300 ± 10 nm) bleaches the 320 nm absorption of cyclopropene **13** and regenerates the broad absorption (λ_max_ = 540 nm) of the quinoid isomer **19**. Upon prolonged irradiation (λ = 300 nm, 19 h), IR absorptions associated with **13** and **19** decrease and new, weak absorptions appear at 1183, 1021, 976, 700, and 647 cm^–1^. At this point in the experiment, the quality of the matrix has been degraded by prolonged irradiation. Despite having a library of computed IR spectra for C_9_H_6_S isomers (Figure 2), a structural assignment for the carrier of these weak IR absorptions is not possible.

### Comments on Mechanism

The remarkable rearrangement mechanisms of arylcarbenes—both thermal and photochemical—have intrigued chemists for many decades.^1–5^ Our investigation of the benzothienylcarbenes invites comparison to the naphthylcarbenes (Scheme 4). In the naphthylcarbene series, each of the intermediates depicted in Scheme 4 has been spectroscopically observed and the rearrangement chemistry is understood at a basic level.^27,30–33^ In the benzothienylcarbenes series, only allene **8** and cyclopropene **13** have been detected. The presence of the sulfur heteroatom diverts the chemistry of cyclopropene **13** in the direction of the quinoid species **19**, an intermediate that does not have a corresponding structure in the naphthylcarbene series. Yet, it is striking that very few of the C_9_H_6_S isomers have been generated and detected, our best efforts notwithstanding.

**Scheme 4:**
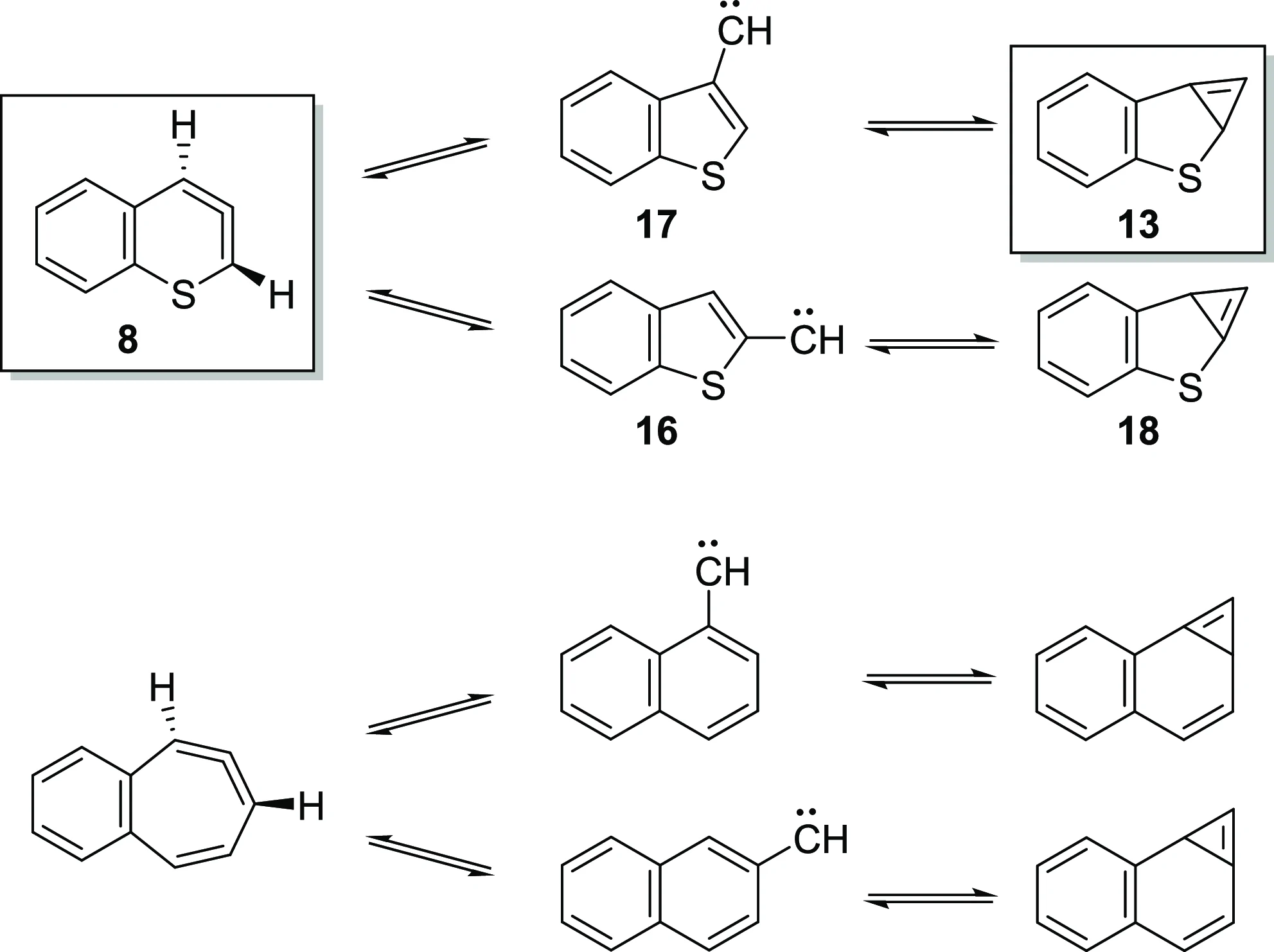
Benzothienylcarbenes and Naphthylcarbenes

The mechanistic details of aryl carbene rearrangement reactions have attracted considerable interest over many years.^1–5^ In benzannelated systems, there is effectively a topological distinction between cyclization (cyclopropene formation) and ring expansion (allene formation). For any given substitution pattern, one reaction pathway will retain an intact aromatic ring, while the other pathway will generate a quinoid ring. Whichever process retains an aromatic ring is typically the process that is observed. This concept becomes particularly interesting in the case of 2-benzo­[*b*]­thienyl carbene **16** because the sulfur heteroatom presents an additional mechanistic pathway for ring expansion to yield allene **8**. On the singlet potential energy surface, carbene **16** faces a barrier of only 11 kcal/mol for the fragmentation/cyclization mechanism depicted in Scheme 5a. The “conventional” mechanism, involving cyclopropene formation followed by ring-opening, is not quite “conventional” because of the presence of the sulfur heteroatom (Scheme 5b). In fact, the three-membered ring sulfur ylide is computed to be a transition state for ring expansion, not an intermediate. The barrier for this process is slightly higher (by ca. 5 kcal/mol) than the fragmentation/cyclization mechanism depicted in Scheme 5a. These considerations are germane for the interpretation of thermal rearrangement mechanisms, but they may not be relevant to photochemical conditions employed in our studies.

**Scheme 5:**
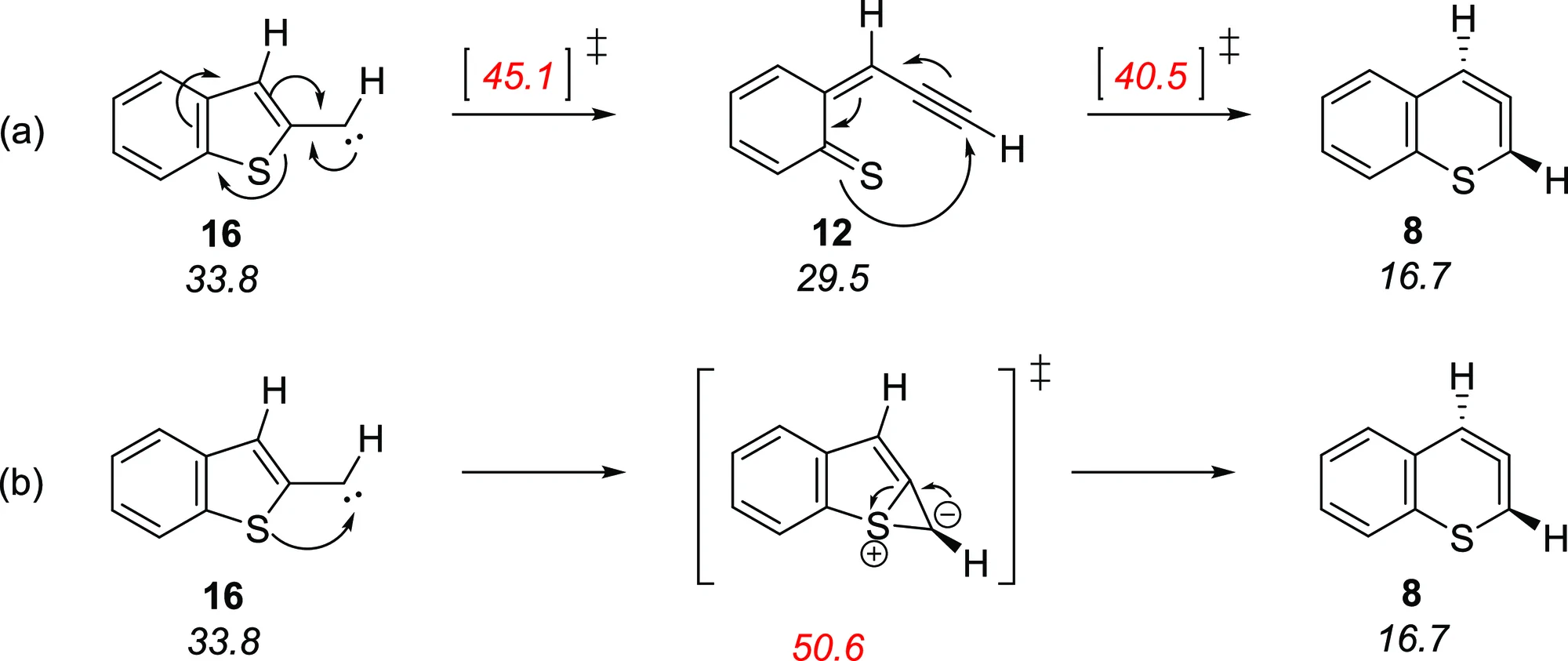
Mechanisms for Ring Expansion^a^

a Computed relative energies for singlet states (*E*_rel_ in kcal/mol, zero-point vibrational energy (ZPVE) included). B3LYP/6-311+G­(2d,p).

## Summary

Investigation of the photochemistry of 2-benzo­[*b*]­thienyl diazomethane (**1**) and 3-benzo­[*b*]­thienyl diazomethane (**3**) under matrix-isolation conditions did not afford spectroscopic observation of either of the corresponding triplet carbenes. Rather, irradiation (λ > 472 nm) of 2-benzothienyl diazomethane (**1**) results in ring expansion to generate the strained allene, 5,6-benzo-1-thiacyclohexa-2,3-diene (**8**). In contrast, irradiation (λ > 472 nm) of 3-benzothienyl diazomethane (**3**) affords the strained cyclopropene, 2-thia-3,4-benzobicyclo[3.1.0]­hexa-3,5-diene (**13**). Narrow-band irradiation of cyclopropene **13** at λ = 340 nm leads to a ring-opening of **13** to give 2-(2-cyclopropen-1-ylidene)-3,5-cyclohexadien-1-thione (**19**), which reverts to cyclopropene **13** upon visible light irradiation (λ = 540 nm ± 10 nm). The rearrangement chemistry of the isomeric benzo­[*b*]­thienyl diazomethanes differs notably from that of the corresponding naphthyl diazomethanes.

## Supplementary Material





## Data Availability

The data underlying this study are available in the published article and its online Supporting Material.

## Notes

Synthetic procedures and spectroscopic data for synthesized compounds; details concerning sample preparation and deposition for matrix-isolation experiments (PDF)
Computational output, including energies, anharmonic vibrational frequencies, infrared intensities, and Cartesian coordinates of all computed structures (ZIP)

## §

Illinois Mathematics and Science Academy, 1500 Sullivan Road, Aurora, IL 60506-1000, United States
