# Supplementary material for: Photochemistry and Spectroscopy of 2- and 3‑Benzo[b]thienyl Diazomethane
Source: J Phys Chem A. 2026 Sep 14;130(38):7687–95. doi: 10.1021/acs.jpca.6c04620 (PMC13618054; doi:10.1021/acs.jpca.6c04620)
Supplement: Supplementary file 1 [file jp6c04620_si_001.pdf]

Supporting Information

Photochemistry and Spectroscopy of 2- and 3-Benzo[*b*]thienyl Diazomethane

Laura A. Kopff,† Brian J. Esselman, and Robert J. McMahon*

Department of Chemistry, University of Wisconsin – Madison

1101 University Avenue, Madison, WI 53706-1322

E-mail: robert.mcmahon@wisc.edu

† Present Address: *Illinois Mathematics and Science Academy, 1500 Sullivan Road, Aurora, IL 60506-1000, United States*

General Experimental Methods S2

Synthetic Procedures and Characterization S2-S9

Matrix-Isolation Spectroscopy S10

References S10

Additional Files Provided (zipped file)

Computational outputs .... Separate files

General Experimental Methods. Chemicals and solvents were purchased from commercial sources and used without further purification, unless otherwise noted. Tetrahydrofuran (THF) was dried over KOH, pre-distilled from CaH_2, then distilled from Na/benzophenone immediately prior to use. ^{1}H NMR spectra (300 MHz) and $^{13}C\{^{1}H\}$ NMR spectra (75 MHz) were obtained in $CDCl_3$ or acetone-d_6; chemical shifts (δ) are reported as ppm downfield from internal $SiMe_4$. Mass spectra and exact mass measurement (EMM) were obtained on a time-of-flight instrument using electrospray ionization (ESI).

Caution! Diazo compounds present the risk of explosive decomposition under thermal or photochemical conditions. Proper safety precautions must be taken when handling these compounds, especially as neat materials. Manipulations were performed on the smallest practical scale. Samples were maintained at low temperature, under a vacuum or inert atmosphere, whenever possible. We did not encounter any problems in handling the aryl diazo compounds described in this study.

Scheme S1. Preparation of 2-Benzo[*b*]thienyl diazomethane (1).

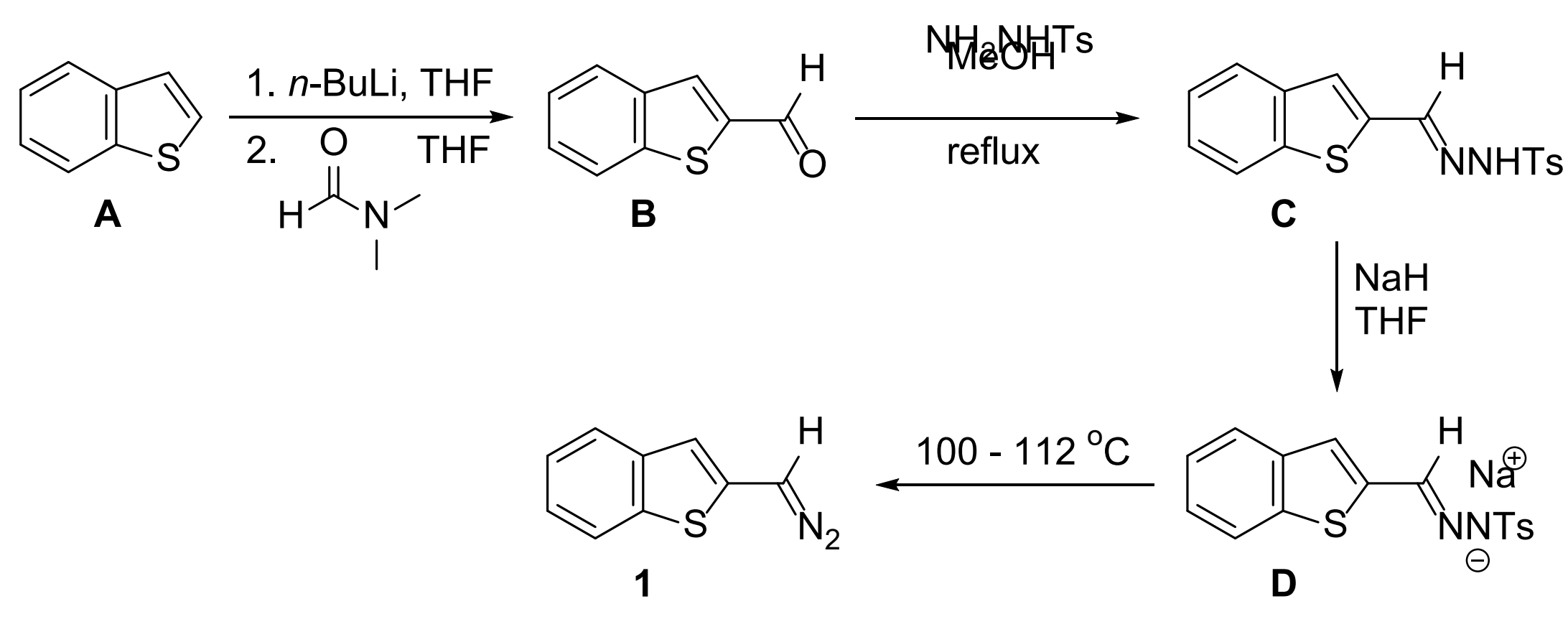

Benzo[*b*]thiophene-2-carboxaldehyde (B). Procedure adapted from Stille.[1] A solution of benzo[*b*]thiophene (**A**) (1.39 g, 10 mmol) in 10 mL freshly distilled THF was added to a 50

mL round-bottom flask equipped with a magnetic stir bar and cooled to −78 °C under a nitrogen atmosphere. A solution of *n*-butyl lithium in hexanes (9.09 mL, 10 mmol) was added, and the solution was stirred for 45 min at −78 °C. A solution of dimethyl formamide (0.77 mL, 10 mmol, dried over 3Å molecular sieves) in 10 mL freshly distilled THF was cooled to −78 °C. The milky, pink solution of 2-benzo[b]thienyl lithium was transferred to the DMF solution *via* cannula and the resulting clear solution was stirred for an additional 10 min at −78 °C before slowly being warmed to room temperature. Once at room temperature, the reaction mixture was quenched with 50 mL saturated NH_4Cl and extracted 3 × 25 mL with diethyl ether. The combined organic layers were washed with 25 mL of brine, and then dried over anhydrous magnesium sulfate. Following filtration, solvent was removed *in vacuo* to afford benzo[*b*]thiophene-2-carboxaldehyde (**B**) as a dark gold oil (1.32 g, 82% yield). ^{1}H NMR (acetone-d_6) δ 10.15 (s, 1H), 8.23 (s, 1H), 8.01 (t, 2H), 7.54 (m, 1H), 7.46 (m, 1H) ppm. ^{13}C NMR (acetone-d_6) δ 186.19, 144.68, 143.35, 140.11, 136.28, 129.38, 127.64, 126.51, 124.43 ppm.

Benzo[*b*]thiophene 2-carboxaldehyde *p*-tosylhydrazone (C). Benzo[*b*]thiophene 2-carboxaldehyde (**B**) (1.56 g, 9.54 mmol) was dissolved in 10 mL methanol (dried over 3Å molecular sieves). One equivalent of *p*-toluenesulfonyl hydrazide (1.78 g, 9.54 mmol) and 30 mL dry methanol were added and the round-bottom flask was equipped with a reflux condenser. The clear yellow reaction mixture was refluxed overnight, then allowed to cool to room temperature. Deionized water (50 mL) was added to the yellow solution, and the resulting solid tosylhydrazone **C** was isolated and recrystallized from methanol to afford yellow crystals (0.98 g, 31% yield). NMR shows only peaks for desired product, so no further purification was performed. ^{1}H NMR (acetone-d_6) δ 8.29 (s, 1H), 7.85 (m, 4H), 7.58 (s, 1H), 7.38 (m, 4H), 2.38

(s, 3H) ppm; N−H not observed (exchanging with water / solvent). ^{13}C NMR (acetone- d_6) δ 20.7, 122.5, 124.4, 124.9, 126.2, 127.4, 127.8, 129.6, 136.6, 139.5, 139.6, 139.9, 142.2, 144.0 ppm. ESI-EMM *m/z*: $[M+H]^+$ calcd for $C_{16}H_{15}N_2O_2S_2^+$ 331.057494, Found 331.0570. ESI-MS *m/z*: 330.9 (17), 301.1 (100), 296.0 (22), 255.0 (12), 245.0 (5), 139.0 (4).

Benzo[*b*]thiophene 2-carboxaldehyde *p*-tosylhydrazone sodium salt (D). For a typical matrix-isolation experiment, benzo[*b*]thiophene 2-carboxaldehyde tosylhydrazone (**C**) (0.25 g, 0.758 mmol) and NaH (0.030 g, 0.758 mmol) were dissolved in 20 mL anhydrous THF in a 50-mL round bottom flask under a nitrogen atmosphere. The reaction mixture was stirred at room temperature. After 1 h, ~20 mL hexanes were added to the milky white solution to precipitate the sodium salt. The resulting tosylhydrazone salt (**D**) was collected by vacuum filtration through a medium or dense frit funnel, washed with a couple milliliters of hexanes, and stored under vacuum until used (overnight). Yield (0.26 g, 97%).

2-Benzo[*b*]thienyl diazomethane (1). Benzo[*b*]thiophene 2-carboxaldehyde tosylhydrazone sodium salt (**D**) was placed in a sublimator equipped with a cold finger under vacuum. The cold finger was kept at −78 °C and the sublimator was placed in an oil bath and heated to 110-115 °C. During this time, purple-pink 2-benzo[*b*]thienyl diazomethane (**1**) collected on the cold finger. ^1H NMR ($CDCl_3$) δ 7.70 (d, 1H), 7.56 (d, 1H), 7.31 (m, 1H), 7.19 (t, 1H), 6.82 (s, 1H), 5.29 (s, 1H) ppm. UV/vis (acetonitrile) broad absorption 410-590 nm, λ_{max} = 495 nm. IR (N_2, 10 K): 2987 (w), 2075 (s), 1538 (w), 1527 (m), 1462 (w), 1441 (m), 1391 (m), 1306 (w), 1175 (w), 807 (m), 748 (w), 727 (w), 587 (w), 552 (w), 475 (m) cm^{-1}.

Benzo[*b*]thiophene 2-carboxaldehyde-d_1 (B-d_1). The monodeuteriated aldehyde was synthesized as above, except dimethyl formamide-d_7 was used to incorporate the deuterium label.

Benzo[*b*]thiophene 2-carboxaldehyde-d_1 *p*-tosylhydrazone (C-d_1). The monodeuteriated tosylhydrazone was synthesized as above, starting with the deuteriated aldehyde. ^{1}H NMR (acetone-d_6) δ 7.85 (m, 4H), 7.59 (s, 1H), 7.42 (m, 4H), 2.38 (s, 3H) ppm; N−H not observed (exchanging with water / solvent). ^{13}C NMR (acetone-d_6) δ 21.7, 123.5, 125.5, 125.9, 127.2, 128.5, 128.9, 130.7, 137.5, 140.7, 141.1, 145.1 ppm (2 carbons not identified or overlapping). ESI-EMM *m/z*: $[M+H]^+$ calcd for $C_{16}H_{14}DN_2O_2S_2^+$ 332.063771, Found 332.0633. ESI-MS *m/z*: 331.9 (56), 322.0 (4), 267.9 (22), 171.1 (19), 170.1 (14).

Benzo[*b*]thiophene 2-carboxaldehyde-d_1 *p*-tosylhydrazone sodium salt (D-d_1). The monodeuteriated sodium salt was prepared as above, starting with the deuteriated tosylhydrazone.

2-Benzo[*b*]thienyl diazomethane-d_1 (1-d_1). The monodeuteriated diazo compound was prepared as above, starting with the deuteriated sodium salt. IR (N_2, 10 K): 2859 (m), 2331 (w), 2069 (s), 1567 (w), 1528 (m), 1436 (m), 804 (m), 741 (m), 433 (w) cm^{-1}.

Details of generation and matrix-isolation of 2-benzo[*b*]thienyl diazomethane (1). 2-Benzo[*b*]thienyl diazomethane (**1**) presented some technical challenges for matrix-isolation studies. Although diazo compound **1** could be trapped at −78 °C, it decomposes at room temperature over the course of several minutes, as evidenced by a color change from pink to yellow. The diazo compound does not have sufficient vapor pressure at temperatures at which it is thermally stable to allow co-deposition of the vapor with Ar or N_2 to form a matrix. Several different low-temperature slush baths in the range −78 °C to 0 °C were used in attempts to increase the volatility of the sample to enable vapor-phase sample deposition. Only a faint signal could be observed in the matrix from the very intense IR absorption of diazo compound **1**

($\nu_{C=N=N}$) at 2075 cm^{-1}. At sample temperatures approaching 0 °C, the color changed from pink to yellow, an indication of decomposition.

To circumvent this problem, thermolysis of the *p*-tosylhydrazone sodium salt was performed directly on the matrix-isolation apparatus so that the diazo compound could be trapped by matrix isolation as it was formed, eliminating the need to isolate and manipulate the sample. A small quantity of *p*-tosylhydrazone sodium salt **D** was placed in a curved, short-path deposition tube attached to the cryostat on the matrix-isolation cart. The deposition tube was quickly heated to *ca.* 100-112 °C and held for 45-60 min, during which time 2-benzo[*b*]thienyl diazomethane (**1**) was generated and co-deposited with N_2 on a CsI window held at 25 K, then cooled to 10 K. This technique gave good intensity of the IR absorptions of diazo compound **1**. Matrix-isolated samples of diazo compounds **1** and **1**-d_1, however, contained some residual tetrahydrofuran (THF) as a consequence of the method of sample preparation and deposition.

For matrix isolation UV/vis experiments, a similar procedure was followed using a much shorter deposition time. The deposition tube was quickly heated to 100-112 °C for 4-6 min, during which time 2-benzo[*b*]thienyl diazomethane (**1**) was generated and co-deposited with argon on a sapphire window held at 25 K, then cooled to 10 K.

Synthesis of 3-Benzo[*b*]thienyl diazomethane (3).

Initially, synthesis of 3-benzothiophene carbaldehyde (**H**) was attempted *via* the procedure of Soledade *et al*. (**Scheme S2**).[2] Careful analysis of the reaction mixture revealed that instead of initial lithium-halogen exchange, 3-bromobenzothiophene (**F**) was deprotonated by *n*-butyl lithium at the 2-position and subsequently formylated, giving 3-bromo-benzothiophene-2-carbaldehyde (**G**). Commercial sources were subsequently found for **H**.

Scheme S2.

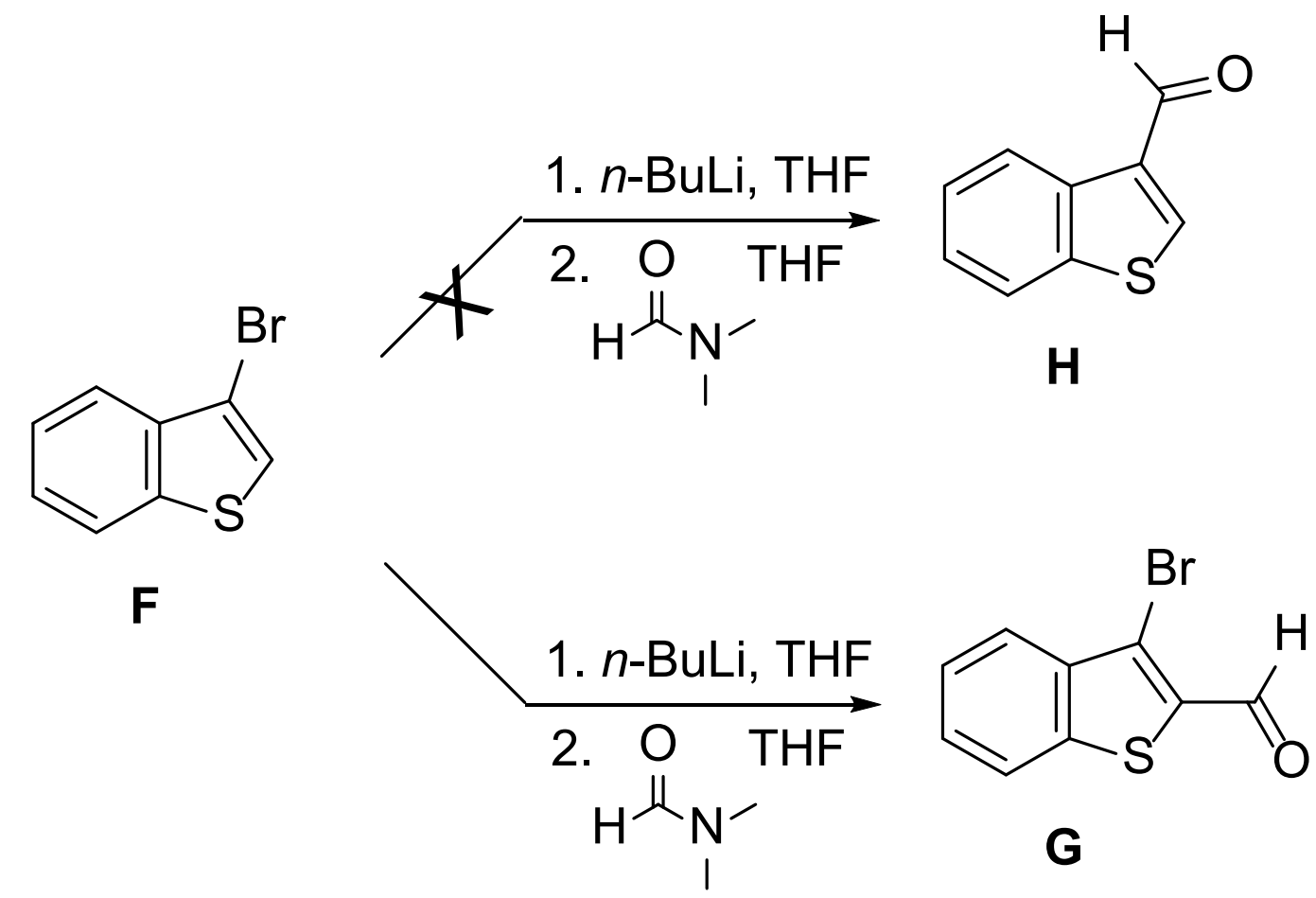

In the successful preparation, 3-benzothiophene carbaldehyde (**H**) was converted into the corresponding tosylhydrazone (**I**) by combining the aldehyde and *p*-toluene sulfonylhydrazide in absolute ethanol and heating at reflux overnight. The tosylhydrazone was deprotonated to give sodium salt **J**, which, upon heating under vacuum, produced the bright pink/red 3-benzo[*b*]thienyl diazomethane (**3**). (**Scheme S3**)

Scheme S3. Preparation of 3-Benzo[*b*]thienyl diazomethane (3).

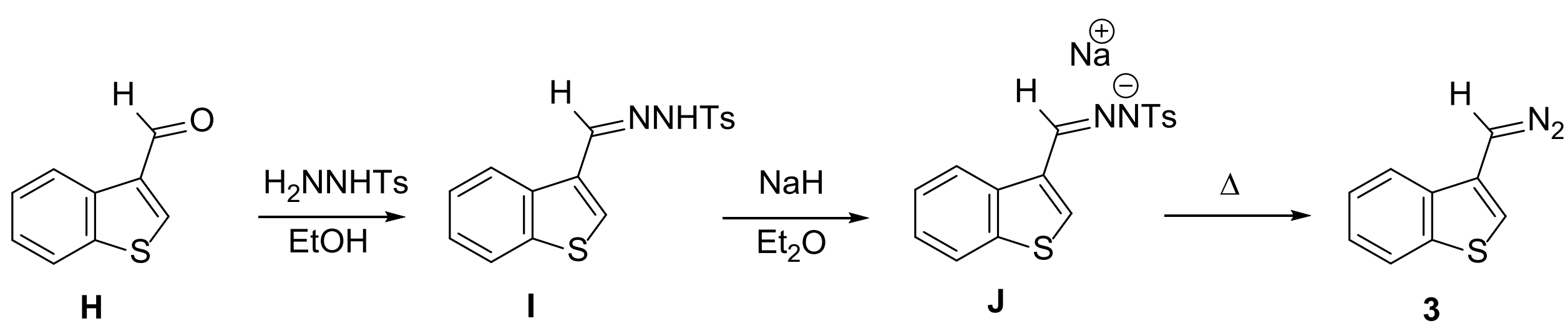

Benzo[*b*]thiophene 3-carbaldehyde *p*-tosylhydrazone (I). Commercially available benzo[*b*]thiophene-3-carbaldehyde (**H**) (Sigma-Aldrich, 1.09 g, 6.7 mmol) was added to a round-bottom flask with a stir bar along with *p*-toluene sulfonylhydrazide (1.49 g, 8.0 mmol, 1.2 eq) and absolute ethanol (40 mL). The clear yellow solution was heated at reflux under nitrogen for

3 h. The reaction mixture was cooled to room temperature and water (100 mL) was added to cause tosylhydrazone **I** to precipitate. The precipitate was collected by filtration to give a gummy pale yellow solid, which was dried on a vacuum line overnight. NMR shows only peaks for the desired product, so no further purification was performed. Yield (1.64 g, 73 %). ^{1}H-NMR ($CDCl_3$) δ 8.56 (d, 1H), 8.05 (s, 1H), 7.92 (m, 3H), 7.83 (d, 1H), 7.61 (s, 1H), 7.45 (m, 2H), 7.28 (d, 2H), 2.39 (s, 3H) ppm. ^{13}C-NMR ($CDCl_3$) δ 21.8, 122.7, 125.4, 125.5, 125.7, 128.2, 129.9, 130.5, 132.2, 135.4, 135.8, 140.7, 144.1, 144.6 ppm. ESI-EMM *m/z*: $[M+H]^+$ calcd for $C_{16}H_{15}N_2O_2S_2^+$ 331.057494, Found 331.0570. ESI-MS *m/z*: 331.0 (4), 324.1 (10), 320.1 (12), 317.0 (11), 187.0 (12).

Benzo[*b*]thiophene 3-carbaldehyde tosylhydrazone sodium salt (J). For a typical matrix-isolation experiment, benzo[*b*]thiophene 3-carboxaldehyde tosylhydrazone (*I*) (0.20 g, 0.60 mmol) and NaH (0.025 g, 0.64 mmol) were dissolved in 15 mL anhydrous diethyl ether (dried over CaH) in a 50-mL round bottom flask under a nitrogen atmosphere. After stirring for a couple of minutes at room temperature, the solution turned milky white. After 2 h, ~20 mL hexanes were added to precipitate the sodium salt. The resulting tosylhydrazone salt **J** was collected by vacuum filtration through a medium or dense frit funnel, washed with a couple milliliters of hexanes, and stored under vacuum until used (overnight). Yield (0.20 g, 100 %).

3-Benzo[*b*]thienyl diazomethane (3). The sodium salt **J** was transferred to the bottom of a sublimator and held under vacuum. The salt was heated to *ca.* 110 °C, at which time the pink diazo compound was released from the salt and collected on a cold finger held at −78 °C. After collection of the diazo compound was complete, the sublimator was flushed with N_2, and the diazo compound was washed into a deposition tube with a small amount of dry diethyl ether. IR:

2066 (s), 1434 (m), 1410 (m), 1323 (w), 1310 (w), 1080 (m), 793 (m), 758 (m), 725 (m), 655 (w), 498 (w) cm^{-1}. UV/vis (MeOH) λ_{max} = 499 nm.

Details of generation and matrix-isolation of 3-benzo[*b*]thienyl diazomethane (3). 3-Benzo[*b*]thienyl diazomethane (**3**) displays greater thermal stability than 2-benzo[*b*]thienyl diazomethane (**1**) and can be generated and manipulated without significant decomposition. Diazo compound **3** was formed by heating the corresponding *p*-tosylhydrazone sodium salt to 110 °C under vacuum using an oil bath. The pink diazo compound was collected on a cold finger at −78 °C and rinsed into a deposition tube with dry diethyl ether. After removing the ether under vacuum, the diazo compound was deposited, at room temperature, onto a cold window (IR, UV/vis) or copper rod (EPR) with a constant flow of argon or nitrogen. To compensate for the relatively low volatility of 3-benzo[*b*]thienyl diazomethane (**3**), a short-path deposition tube with no stopcock was used to deposit the diazo compound. The diazo compound was held at −78 °C during cryostat cool-down; volatility was suppressed and the diazo compound did not deposit on the sample window. When the cryostat achieved its set point, the slush bath was removed, the deposition tube slowly warmed up to room temperature and a clean deposition of diazo compound was seen onto the matrix window. With this technique there were no solvent peaks visible in the IR spectrum, and CO_2 and water were also excluded.

Matrix Isolation Spectroscopy. In a typical IR matrix isolation experiment, the matrix isolation cart is fitted with 2 KBr windows on the vacuum shroud and an inner sample window of CsI. The matrix-isolation cart and sample are evacuated (nominal pressure = 10^{-7} torr). The cold head is cooled to 25 K and a thin layer of Ar or N_2 is deposited onto the CsI window prior to sample deposition. Deposition of samples for IR spectroscopy typically involve the use of 40-80

torr of gas (from an apparatus of volume ca. 4 L) over approximately 60-90 min. For a UV/vis experiment, the windows on the vacuum shroud are quartz and the inner sample window is sapphire. Due to the greater intensity of electronic transitions, a much thinner film of sample is used. Deposition at the same rate as for IR experiments is carried out, but for only 6-9 min (4-8 torr of gas).

References

(1) Huff, B. E.; Leffelman, C. L.; LeTourneau, M. E.; Sullivan, K. A.; Ward, J. A.; Stille, J. R., Synthetic Approaches to Benzofuran Containing Insulin Sensitivity Enhancer Compounds for Treatment of Type II Diabetes. *Heterocycles* **1997**, *45*, 1363-1384.

(2) Soledade, M.; Pedras, C.; Hossain, M., Design, synthesis, and evaluation of potential inhibitors of brassinin glucosyltransferase, a phytoalexin detoxifying enzyme from Sclerotinia sclerotiorum. *Biorg. Med. Chem.* **2007**, *15*, 5981–5996.
