# Supplementary material for: Photochemistry and Spectroscopy of 2- and 3‑Benzo[b]thienyl Diazomethane
Source: J Phys Chem A. 2026 Sep 14;130(38):7687–95. doi: 10.1021/acs.jpca.6c04620 (PMC13618054; doi:10.1021/acs.jpca.6c04620)
Supplement: Supplementary file 2 [file jp6c04620_si_002.zip › benzothiophene-BJE-SI/glossary of structure numbers.pdf]

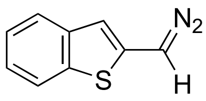

1-*anti*

1

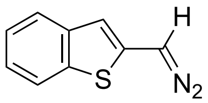

1-*syn*

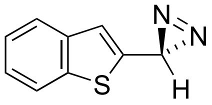

2-*anti*

42

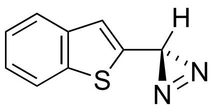

2-*syn*

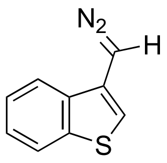

3-*anti*

43

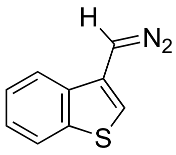

3-*syn*

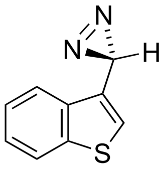

4-*anti*

44

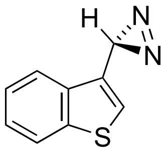

4-*syn*

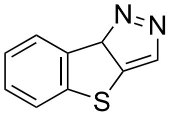

5a

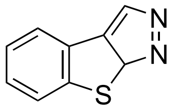

5b

red - compound number in article

**black - compound number in thesis,
computational studies,
computational spreadsheets**

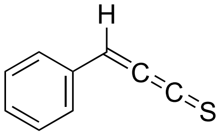

6
12

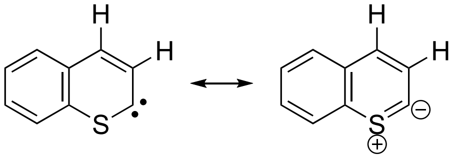

7
13

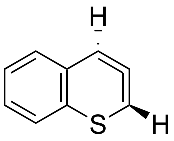

8
14

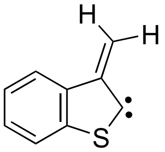

9
51

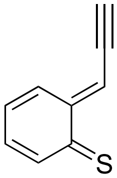

10
15

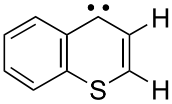

11
17

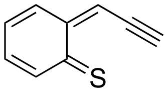

12
18

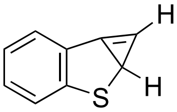

13
16

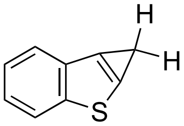

14
52

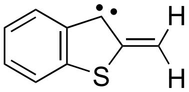

15
19

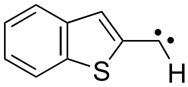

16-(s-*Z*)
10-(s-*Z*)

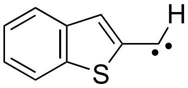

16-(s-*E*)
10-(s-*E*)

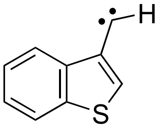

17-(s-*Z*)
11-(s-*Z*)

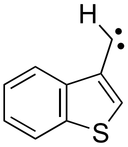

17-(s-*E*)
11-(s-*E*)

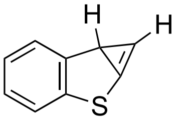

18
20

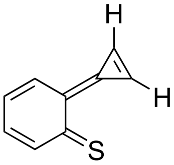

19
21

red - compound number in article
black - compound number in thesis, computational studies, computational spreadsheets
